# Surgical management of ectopic Cushing’s syndrome from a pulmonary carcinoid secreting CRH supported by intraoperative ACTH monitoring – a case report

**DOI:** 10.3389/fendo.2026.1898005

**Published:** 2026-09-23

**Authors:** Agata Pokrzywa, Jan Domanski, Zuzanna Roszkowska, Lukasz Koperski, Dariusz Wasowski, Urszula Ambroziak, Janusz Pachucki, Malgorzata Bobrowicz

**Affiliations:** 1 Department of Internal Medicine and Endocrinology, Medical University of Warsaw, Warsaw, Poland; 2 Doctoral School of Medical University of Warsaw, Medical University of Warsaw, Warsaw, Poland; 3 Department of Pathology, Medical University of Warsaw, Warsaw, Poland; 4 Department of Thoracic Surgery, National Medical Institute of the Ministry of the Interior and Administration, Warsaw, Poland

**Keywords:** ACTH, carcinoid, CRH secretion, ectopic Cushing’s syndrome, intraoperative ACTH monitoring

## Abstract

An intraoperative ACTH monitoring during resection of hormone-secreting neuroendocrine neoplasms remains a subject of a debate. We present the case of a 36-year-old man with clinical and biochemical features of ACTH-dependent hypercortisolism. Dynamic endocrine testing, including the high-dose dexamethasone suppression test, metyrapone test, and desmopressin stimulation test resulted in inconclusive results, precluding clear differentiation between Cushing’s disease and ectopic Cushing’s syndrome. Pituitary imaging revealed no definite focal lesion, whereas chest imaging detected a small pulmonary nodule along with an incidental adrenal adenoma. Taking into account rapid clinical course and the severity of clinical symptoms surgical resection of the lung lesion was undertaken with intraoperative ACTH monitoring. Due to compilation of factors patient was sent to thoracic surgery without delay. A transient ACTH surge was observed during first stage tumor excision and, even though intraoperative pathological and surgical findings indicated the complete tumor resection, which contributed to the decision to extend the procedure to an upper lobectomy, after which a marked and sustained decline in ACTH concentrations was observed. Histopathological examination confirmed a typical pulmonary carcinoid tumor, and surgical treatment resulted in clinical and biochemical remission, followed by gradual recovery of the hypothalamic-pituitary-adrenal axis. This case highlights the limitations of dynamic testing and imaging in ECS, particularly in indolent presentation, and underscored the potential value of intraoperative ACTH monitoring as an adjunctive tool for tumor localization and surgical decision-making.

## Introduction

Cushing’s syndrome (CS) results from supraphysiological exposure to glucocorticoids and is associated with significant morbidity and mortality. The etiology is either exogenous from glucocorticoid administration or endogenous due to autonomous overproduction of cortisol. Approximately 10–20% of endogenous cases are due to ectopic Cushing’s syndrome (ECS), caused by adrenocorticotropin (ACTH)–producing tumors outside the pituitary gland (1, 2). In rare cases, ECS may be caused by corticotrophin-releasing hormone (CRH) secreting tumors (3, 4). ECS characteristically presents with rapidly progressive hypercortisolism, and timely identification of the ACTH source is critical for preventing life-threatening complications. Optimal outcomes depend on a systematic strategy that emphasizes rapid diagnosis, accurate tumor localization, control of cortisol excess, and, when feasible, surgical removal of the underlying tumor (5, 6). However, dynamic endocrine tests and imaging findings may be discordant or misleading, especially in indolent ECS, making timely localization challenging. This case illustrates such diagnostic pitfalls and highlights the role of intraoperative ACTH monitoring in the management of ECS. Extensive diagnostic workup might delay the appropriate surgical resection of the potentially malignant neuroendocrine tumor and pose additional risk due to excess cortisol exposure or invasive diagnostic procedure complications.

## Case report

A 36-year-old man was referred to the Endocrinology Department for evaluation of ACTH-dependent hypercortisolemia identified in the outpatient setting. He reported pronounced muscle weakness, a 15-kg weight gain, progressive facial rounding and erythema, increased hair shedding, acne, and extensive violaceous striae on the abdomen and upper limbs, which progressed over the past 18 months. He denied any prior glucocorticoid therapy.

At admission, the patient was in a relatively stable general condition. Physical examination revealed classic cushingoid features, including a plethoric, rounded face; prominent red striae on the abdomen, arms, and thighs; acneiform eruptions on the trunk and upper limbs; and disproportionate accumulation of adipose tissue in the supraclavicular, cervical, and abdominal regions. Markedly elevated arterial pressure was also noted (180/110 mmHg). **Figure 1** illustrates the progression and intensification of the stigmata of hypercortisolism (B-D), showing patient photographs taken 1.5 years prior to admission and at the time of admission (A).

**Figure 1:**
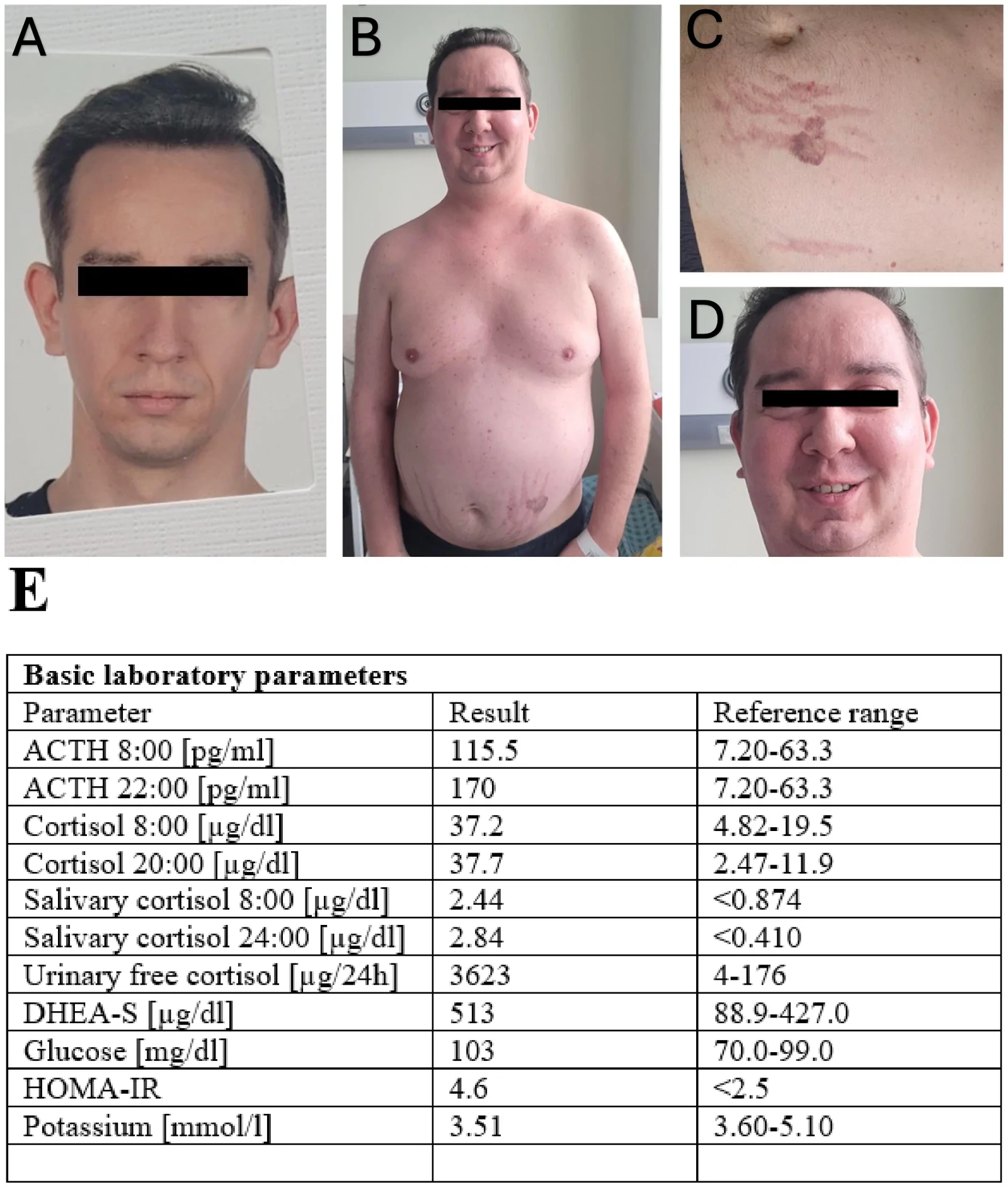
Clinical picture of the patient. Photographs of the patient **(A)** taken 1.5 years before hospital admission demonstrating the absence of overt cushingoid features, and **(B–D)** photographs obtained at admission showing facial rounding, supraclavicular fullness, centripetal adiposity, acneiform eruptions, and extensive violaceous striae. **(E)** Laboratory parameters at admission.

Biochemical testing confirmed hypercortisolemia with urinary free cortisol (UFC) levels approximately 20-fold above the upper limit of normal and loss of diurnal rhythm of serum and salivary cortisol. Potassium levels were mildly decreased.

For differential diagnosis and determining the etiology of CS, the patient subsequently underwent a comprehensive set of functional laboratory assessments and imaging studies. The dynamic testing consisted of high-dose dexamethasone suppression test (HDDST), overnight single-dose metyrapone test and desmopressin stimulation test (**Table 1**).

**Table 1:** Results of dynamic testing: high-dose dexamethasone suppression test (HDDST), overnight single-dose metyrapone test and desmopressin stimulation test.

High-dose dexamethasone suppression test (HDDST) and overnight single-dose metyrapone test					
Parameter	Baseline	1 mg DST	4 mg DST	8 mg DST	Metyrapone
Cortisol [µg/dl]	**30.1**	37.1	24.3	**18.6**	**5.88**
Urinary free cortisol [ug/24h]	**3623**	2683	884	**130**	X
ACTH [pg/ml]	**115.5**	163	82.2	**115**	**154**
Desmopressin stimulation test					
Time [min.]	Cortisol [µg/dl]	ACTH [pg/ml]			
-10’	8.17	**152.0**			
0’	**8.04**	191.0			
+10’	8.25	**219.0**			
+15’	8.66	**219.0**			
+30’	8.84	150.0			
+45’	**9.08**	182.0			

Minimum and maximum values are highlighted in bold type.

In the 8 milligram HDDST there was a 11.5 ug/dl (around 38%) decrease in serum cortisol, a 3493 ug/dl (around 96%) decrease in UFC and 0.5 pg/ml (around 0.5%) decrease in ACTH concentration, which did not clearly support the diagnosis of Cushing’s disease (CD) (7).

In our center, the overnight single-dose metyrapone test has historically been used as an adjunctive test in the differential diagnosis of ACTH-dependent Cushing’s syndrome. Given its limited contemporary validation, variability in testing protocols, and variable diagnostic performance, its result was interpreted only as supportive information rather than as a definitive discriminator between pituitary and ectopic ACTH secretion (8). In our patient, cortisol decreased by approximately 80%, while ACTH increased by approximately 33%, indicating some preservation of corticotroph responsiveness; however, this finding was not considered sufficient to establish a pituitary origin of the disease.

The desmopressin stimulation test, which involves bolus administration of desmopressin (8 ug in our center) is used to detect ACTH-secreting pituitary adenomas based on the expression of vasopressin V2/V3 receptors by corticotroph pituitary tumors and presumed absence of those cells in tumor ectopically secreting ACTH (9). In our patient there was 1.04 ug/dl (around 13%) increase in cortisol and 66 pg/ml (around 44%) increase in ACTH concentration. These findings represented a borderline response, with an increase in ACTH but only a modest increase in cortisol, and were therefore considered partially suggestive of Cushing’s disease rather than diagnostic (9).

Given the discordant results of the dynamic tests, contrast-enhanced pituitary MRI was performed but revealed no definite focal lesion (**Figure 2**). Given the discordant results of dynamic testing and the absence of a definite pituitary lesion on MRI, bilateral inferior petrosal sinus sampling (BIPSS) would have represented an appropriate additional diagnostic procedure. However, BIPSS was not performed because of its limited local availability and lack of timely access to an experienced referral center. In the presence of a distinct contrast-enhancing pulmonary lesion considered a plausible ectopic source, the multidisciplinary team elected to proceed with surgical exploration of the lung lesion. Abdominal MRI and chest CT (**Figure 2**) revealed a left adrenal nodule consistent with an adenoma and an 8 × 6 mm enhancing nodule in the left lung. 68Ga-DOTATATE PET/CT scan showed no pathological uptake.

**Figure 2:**
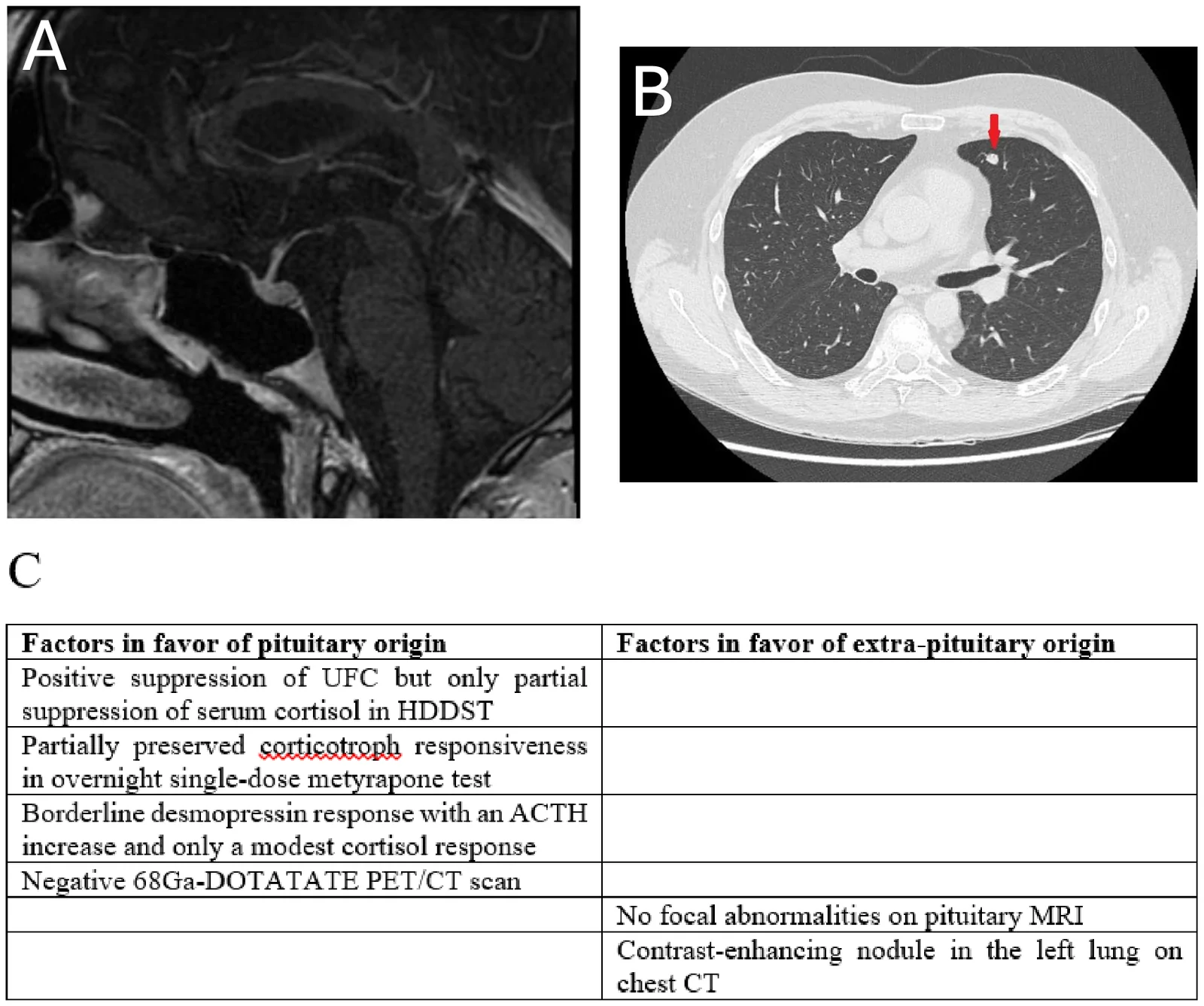
Additional imaging studies. Pituitary magnetic resonance **(A)** showing no focal abnormalities, chest computed tomography scan **(B)** showing an irregular, contrast-enhancing 8 × 6 mm nodule in segment 3 of the left lung. **(C)** Evidence supporting pituitary or extra-pituitary source of CS in this patient.

The attending physician decided not to delay surgical treatment by initiating any medical therapy, including cortisol synthesis inhibitors or ACTH-directed therapy. Therefore, the ^68Ga-DOTATATE PET/CT scan was performed without prior medical treatment. It should be taken into account that severe hypercortisolism has been shown to downregulate somatostatin receptor subtype 2 (SSTR2) expression, potentially reducing the sensitivity of somatostatin receptor imaging. In view of the clinical, laboratory and imaging data the decision was made to apply visible tumor resection strategy, assuming that the lung lesion is the source of the hormonal excess. However, given this uncertainty, intraoperative ACTH level monitoring was performed to support the diagnosis.

The patient initially underwent minimally invasive thoracoscopic wedge resection encompassing the suspicious lesion and adjacent parenchyma in segment 3. ACTH concentrations were measured preoperatively and every 30 minutes intraoperatively (**Figure 3**). Notably, a marked ACTH surge occurred at minute 90, immediately after excision of the segment 3 fragment. Frozen-section examination at that time showed a 0.8-cm lesion compatible with a neuroendocrine tumor, situated 0.6 cm from the resection margin. Consequently, the procedure was extended to an upper lobectomy, during which ACTH levels demonstrated a steep and continuous decline. The postoperative course was uncomplicated, and the patient was discharged after several days with instructions for gradual tapering of hydrocortisone replacement therapy. Definitive histopathology confirmed a typical carcinoid tumor of the lung (pT1aN0MXR0). A total of 7 lymph nodes were examined, all free from the metastases (0/7). Immunohistochemistry showed positivity for synaptophysin and chromogranin A, with a Ki-67 proliferation index of approximately 1%. Additional staining revealed a partial positive reaction for CRH and a negative reaction for ACTH. After removal of the automatic stapler line, a second microscopic tumor focus was identified within the concealed surgical margin. The temporal association between this finding and the intraoperative ACTH surge raises the possibility that residual tumor tissue contributed to the observed hormonal pattern; however, a direct causal relationship cannot be established. Given the partial CRH immunoreactivity and negative ACTH staining, alternative explanations include hormone release associated with tumor manipulation, transient CRH-mediated stimulation of pituitary ACTH secretion, and the physiological neuroendocrine response to surgical stress.

**Figure 3:**
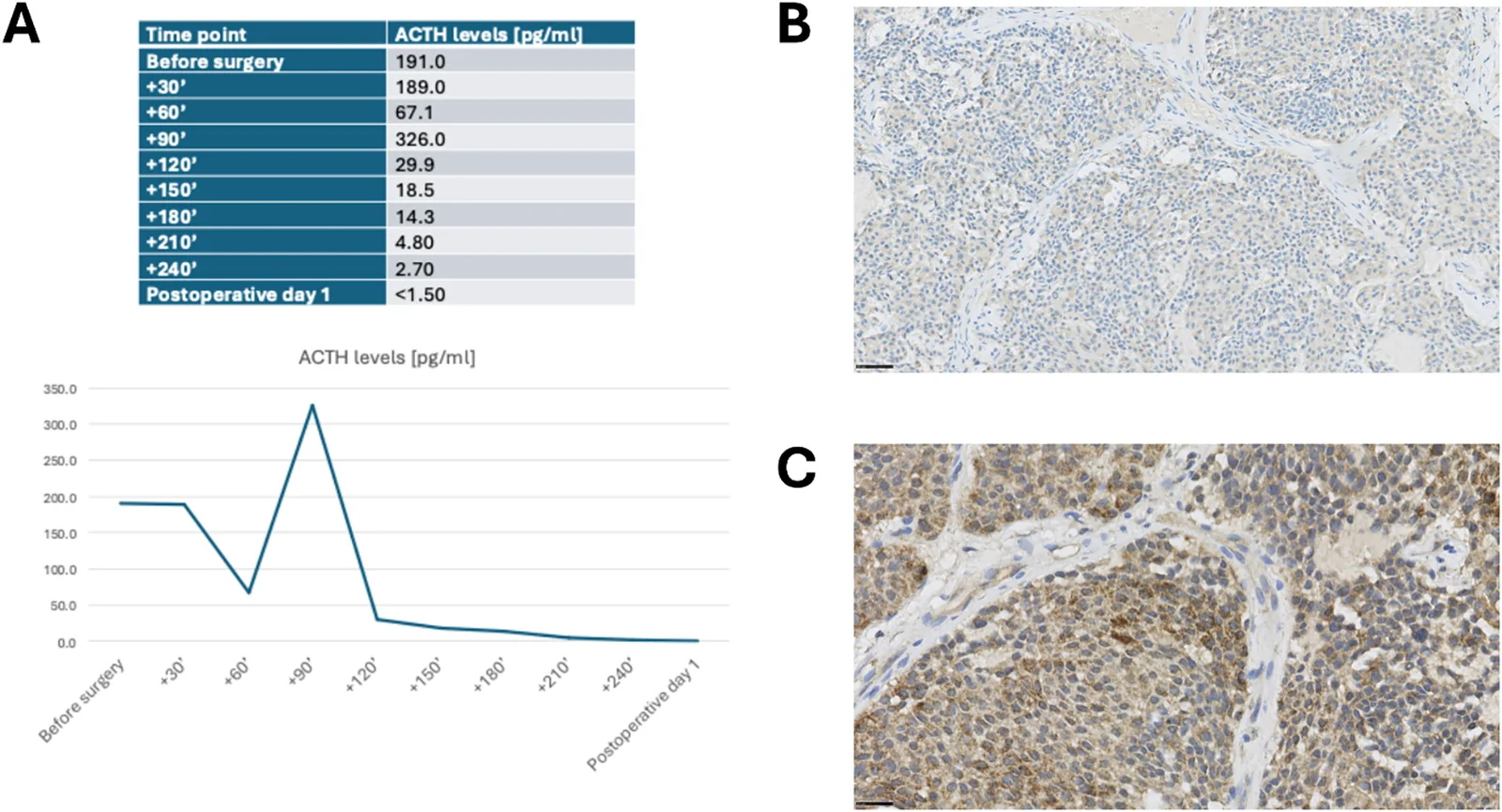
**(A)** Intraoperative ACTH concentrations with a sudden rise in ACTH concentration at 90 minutes during the procedure. **(B, C)** Histopathology - pulmonary carcinoid tumor. Immunohistochemical stain for **(B)** ACTH, scale bar: 50 μm, **(C)** Immunohistochemical stain for CRH, scale bar: 50 μm.

Surgical treatment resulted in clinical and biochemical remission of CS (**Figures 4A, B**). In the subsequent weeks the patient experienced a 7-kg weight loss and normalization of arterial blood pressure, allowing for discontinuation of antihypertensive medications.

**Figure 4:**
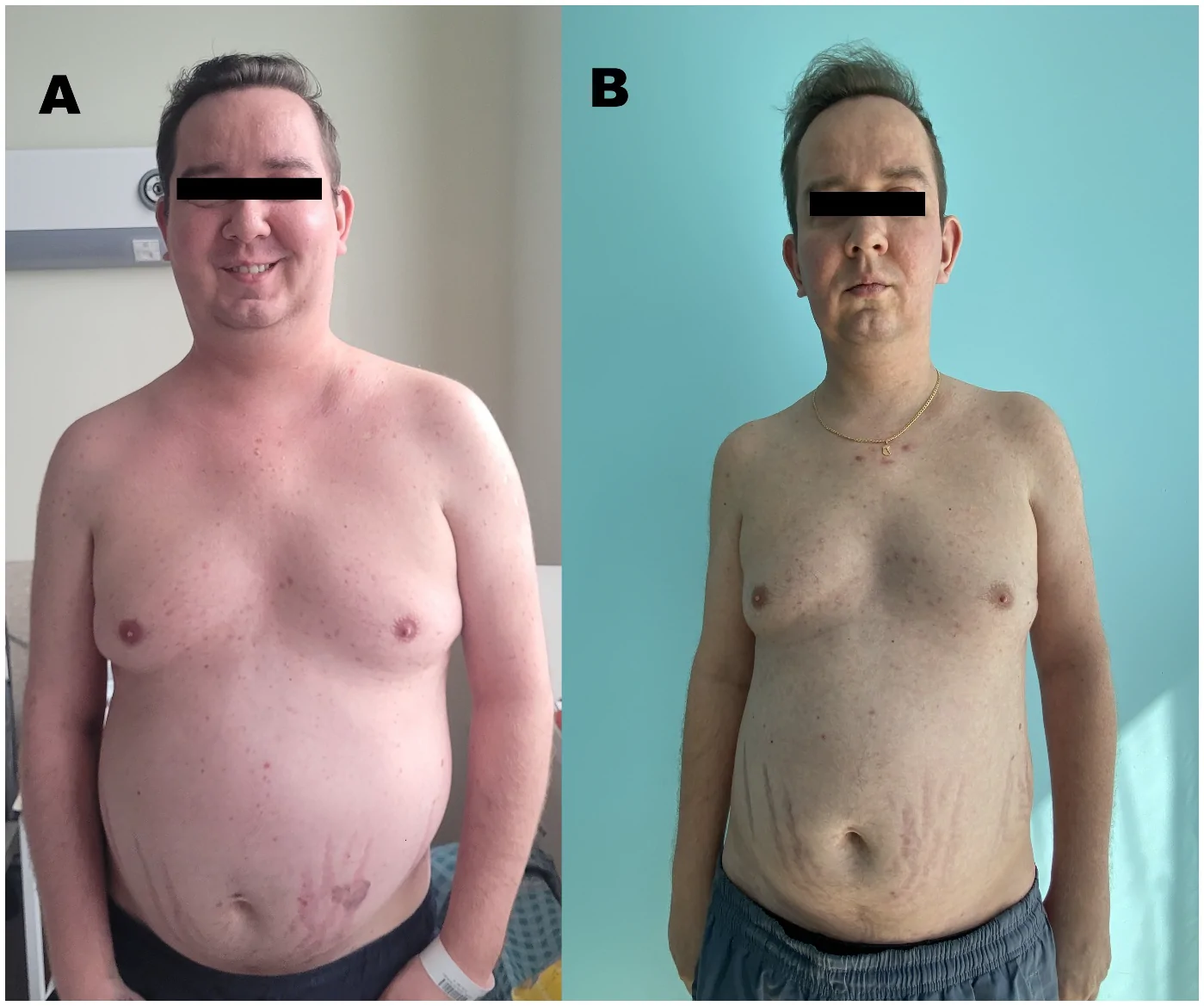
Photographs of the patient **(A)** at admission **(B)** 2 months after surgical intervention.

Initially biochemical features of adrenal insufficiency were seen with low serum cortisol, ACTH, and DHEA-S concentrations (2 months after surgery – morning serum cortisol of 1,79 ug/dl, ACTH 26,66 pg/ml, DHEAS 29,24 ug/dl), followed by progressive normalization of parameters consistent with the gradual recovery of the patient’s adrenal function, allowing for discontinuation of hydrocortisone replacement therapy after 3 months. At the evaluations conducted 15 and 21 months after surgical treatment, neither clinical assessment nor biochemical testing with the 1 mg dexamethasone suppression test indicated recurrence of hypercortisolemia (cortisol in DST: 1,17 and 0,58 ug/ml). From the patient’s perspective, he felt relieved after tumor resection and was able to return to his previous lifestyle. He remains grateful for the treatment and reports a sustained improvement in his well-being and quality of life. He stays in regular contact with his endocrinologist and was willing to share his story.

## Discussion

ECS, first described by Liddle in 1962 (10), results from ACTH/CRH secretion by non-pituitary tumors and typically presents with rapidly progressive hypercortisolism. ECS is classified into “aggressive” and “indolent” forms. “Indolent” ECS, like the one presented-most commonly caused by bronchial carcinoids-often evolves more slowly and may closely mimic CD, creating substantial diagnostic difficulties (5, 11, 12). Neuroendocrine neoplasms (NEN) of the thorax and upper abdomen account for most ECS cases, with bronchial NENs representing the most frequent source (5, 6, 13, 14).

According to current experts’ opinion, the diagnostic evaluation of ACTH-dependent CS should follow a structured, stepwise approach. The process begins with confirmation of endogenous hypercortisolism using at least two first-line tests, such as the overnight 1-mg dexamethasone suppression test, late-night salivary cortisol, or 24-hour urinary free cortisol (15). Once inappropriate cortisol production is established, plasma ACTH measurement determines whether the hypercortisolism is ACTH-dependent. If ACTH dependence is confirmed, dynamic endocrine tests-most commonly the CRH stimulation test, which is currently unavailable, or high-dose dexamethasone suppression test-are used to differentiate pituitary from ectopic ACTH secretion (15). A stepwise diagnostic approach to patients with suspected CS has been elegantly summarized by Ragnarsson et al. (2).

In our patient, several diagnostic pitfalls complicated the evaluation. Dynamic testing yielded partially pituitary-like but non-diagnostic responses, including partial suppression during the high-dose dexamethasone suppression test and a borderline response to desmopressin. The overnight single-dose metyrapone test, which is used locally as an adjunctive test, also demonstrated preserved corticotrophin responsiveness; however, given its limited validation as a discriminator between pituitary and ectopic ACTH secretion, this result was not considered a major argument in favor of Cushing’s disease (16, 17). Furthermore, a negative 68Ga-DOTATATE PET/CT argued against an ectopic source; however, given the small size of the pulmonary nodule and downregulation of SSTR2 expression by cortisol, false-negative functional imaging cannot be entirely excluded, and ECS therefore remained a theoretical possibility (18).

Recently, personalized non-invasive diagnostic algorithms incorporating the magnitude of urinary free cortisol elevation have been proposed for differentiating pituitary from ectopic ACTH-dependent Cushing’s syndrome. Lavoillotte et al. demonstrated that markedly elevated UFC concentrations, particularly when exceeding 10-fold the upper limit of normal and accompanied by an identifiable extra-pituitary tumor, may substantially increase the probability of an ectopic source and help guide subsequent localization studies. In our patient, UFC was approximately 20-fold above the upper limit of normal and CT revealed a distinct pulmonary nodule, findings that were therefore strongly suggestive of an ectopic source. Nevertheless, the negative ^68Ga-DOTATATE PET/CT and partially pituitary-like responses to dynamic testing maintained diagnostic uncertainty, illustrating the limitations of relying on individual non-invasive findings in complex cases (19).

The explanation for why bilateral inferior petrosal sinus sampling (BIPSS) was not performed is crucial for understanding the complexity of this ACTH-dependent Cushing’s syndrome case. First, delaying surgical treatment in the setting of a suspected malignancy may have detrimental consequences. The decision to proceed directly with surgical treatment was made by the attending physician based on his clinical experience, the available diagnostic data, and the local healthcare system constraints. In our center, scheduling BIPSS generally takes approximately two months, although the waiting time may vary depending on unpredictable logistical factors.

It is not common for a surgeon to consult an endocrinologist intraoperatively, as fortunately occurred in this case. However, the interpretation of the intraoperative ACTH surge warrants caution. In this case, the decision was made by the surgeon immediately after, or even during, a telephone consultation with the endocrinologist, and the overall risks and benefits were weighed instantaneously by the surgeon. Since the surgery was performed at a different center, we did not retrospectively discuss with the surgeon the correlation between the pathological findings and the preoperative radiological studies.

An interesting aspect of this case are the results of pathomorphological immunostaining suggesting that CRH rather than ACTH may have been the hormone secreted by the tumor. CRH secretion is a very rare cause of ACTH-dependent CS (20) and in such tumors CRH is usually co-secreted with ACTH (21). Isolated CRH secretion is most commonly reported in medullary thyroid carcinomas and phaeochromocytomas, although carcinoids are also implicated as a possible source (22). Patients with CRH secreting tumors typically exhibit more aggressive clinical phenotype, however, in this case, the patient demonstrated a comparatively indolent disease course, with symptoms developing over the course of 1.5 years. In CRH-driven ECS hypercortisolism arises through the activation of the pituitary, which increases ACTH and subsequently drives adrenal cortisol production. As a result, both ACTH and cortisol may show suppression during high-dose dexamethasone testing, mimicking the response seen in pituitary-dependent disease (23). This finding may be a possible explanation for the positive suppression of UFC but only partial suppression of serum cortisol during HDDST in the presented case.

When biochemical and imaging findings are discordant, BIPSS represents an important diagnostic tool for differentiating pituitary from ectopic ACTH secretion. In particular, BIPSS is generally recommended in patients with ACTH-dependent Cushing’s syndrome in whom pituitary MRI is negative or equivocal and non-invasive testing does not provide a conclusive diagnosis. In the present case, BIPSS would therefore have represented an appropriate additional diagnostic step. However, because of its limited local availability and lack of timely access to an experienced referral center, the procedure was not performed. In view of the presence of a distinct contrast-enhancing pulmonary lesion considered a plausible ectopic source, the multidisciplinary team elected to proceed with surgical exploration. We acknowledge that the absence of BIPSS represents a limitation of the diagnostic work-up in this case and that this individualized approach should not be regarded as a substitute for BIPSS in similarly equivocal cases (15).

The value of intraoperative hormonal monitoring in ectopic ACTH syndrome was demonstrated in one of the earliest reports published in 1995 (24). In this five-patient series involving ACTH-secreting bronchial carcinoids, rapid immunochemiluminescent ACTH assays (15-minute turnaround time) enabled real-time assessment of surgical completeness. A rapid fall in arterial ACTH concentration within 15 minutes of tumor excision reliably indicated successful removal of the ectopic source (24). Similar conclusions have been reported in patients with CD. A study involving a cohort of 55 patients with CD demonstrated the usefulness of intraoperative ACTH level monitoring for the prediction of remission (25). In our case, intraoperative ACTH monitoring provided additional real-time information during surgical resection. An unexpected ACTH increase was observed following the initial wedge resection. The abrupt ACTH increase observed following the initial resection should therefore be regarded as an intraoperative signal raising concern regarding possible residual hormonally active tissue rather than as direct evidence of a second ACTH-secreting tumor focus. Subsequent histopathological examination revealed a second microscopic tumor focus within the concealed surgical margin. Thus this technique remains a valuable adjunct in selected patients with suspected multifocal disease, unclear margins, or small pulmonary carcinoids.

The goals of ECS management are to normalize cortisol levels and control tumor growth. Complete surgical removal of the tumor is the only curative option, but this is not always feasible, especially in patients with metastatic NENs or an occult source of ECS. Hypercortisolism can be managed medically with adrenal steroidogenesis inhibitors, such as osilodrostat, which has demonstrated significant effectiveness, achieving normalization of UFC in approximately 40% of patients after only four weeks of therapy (26). Consequently, osilodrostat should be considered a first-line treatment for ECS, particularly in patients with severe hypercortisolism (26), although some reports indicate that ketoconazole, metyrapone or mitotane may be used as well (27, 28). In Poland, access to osilodrostat may remain challenging, primarily because of the high treatment cost and limitations related to reimbursement pathways. Nevertheless, osilodrostat is available and is used in selected specialized endocrine centers experienced in the management of Cushing’s syndrome. Recent Polish real-world evidence further demonstrates the feasibility of individualized osilodrostat treatment in patients with ACTH-dependent Cushing’s syndrome (29). Endoscopic bilateral adrenalectomy has also been reported as a treatment option in ECS, especially in cases where radical resection of the primary tumor is impossible or the primary tumor is unknown (30).

Several limitations of this case should be acknowledged. First, BIPSS was not performed despite discordant biochemical and imaging findings, leaving uncertainty regarding the source of ACTH-dependent hypercortisolism before surgery. This limitation highlights the importance of referring diagnostically challenging cases of Cushing’s syndrome to centers of excellence with timely access to BIPSS and multidisciplinary teams comprising endocrinologists, experienced interventional radiologists, endocrine surgeons or thoracic surgeons, radiologists, nuclear medicine specialists, and pathologists. Such an approach may facilitate appropriate interpretation of discordant findings and reduce diagnostic uncertainty. Second, severe hypercortisolism was not pharmacologically controlled before surgery. Although the decision to avoid delaying resection of a potentially malignant pulmonary lesion was individualized and based on the clinical circumstances, persistent hypercortisolism at the time of surgery may increase perioperative and postoperative morbidity, including cardiovascular, thromboembolic, metabolic, infectious, and wound-related complications. Therefore, whenever clinically feasible, preoperative control of severe cortisol excess should be considered, particularly in specialized centers experienced in the management of Cushing’s syndrome.

This case highlights the diagnostic challenges associated with ECS, particularly when endocrine test results partially mimic those of Cushing’s disease and imaging findings are non-definitive. Intraoperative ACTH monitoring provided complementary real-time information and, together with surgical and pathological findings, contributed to the assessment of resection completeness. The unexpected ACTH increase after initial tumor excision was temporally associated with the subsequent identification of a second microscopic tumor focus, although a direct causal relationship cannot be established. These findings support the potential role of intraoperative ACTH monitoring as an adjunctive tool in selected diagnostically and surgically challenging cases.

## Data Availability

The original contributions presented in the study are included in the article/supplementary material. Further inquiries can be directed to the corresponding author/s.
